# The effects of virtual reality training in stroke and Parkinson’s disease rehabilitation: a systematic review and a perspective on usability

**DOI:** 10.1186/s11556-022-00283-3

**Published:** 2022-01-25

**Authors:** Ksenija Sevcenko, Ingrid Lindgren

**Affiliations:** 1 Department of Health Sciences, Lund University, Lund, Sweden; 2 Department of Neurology, Rehabilitation Medicine, Memory Disorders and Geriatrics, Skåne University Hospital, Lund, Sweden

**Keywords:** Virtual reality exposure therapy, Stroke, Rehabilitation, Parkinson’s disease, Telerehabilitation, Healthy aging

## Abstract

**Background:**

Virtual Reality (VR) training is emerging in the neurorehabilitation field. Technological advancement is often faster than clinical implementation. Previous reviews stressed the study design and methodological weaknesses of research in the field of VR for neurorehabilitation. Clinically relevant conclusions on implementation in particular patient groups are needed.

The aim was to update the existing knowledge with the recent evidence on the effects of VR training on functional ability of patients with stroke and Parkinson’s Disease (PD). Secondary objective was to analyze the aspects of usability of VR intervention in these populations.

**Methods:**

Systematic literature search (via PubMed, CENTRAL) was conducted from inception to February 29, 2020 to identify suitable articles for two population subcategories. Randomized controlled trials published from 2016 to 2020, investigating the effectiveness of VR on a variety of outcomes contributing to the functional independence were included. Critical Appraisal Skills Programme (CASP) checklist was used for a methodological quality assessment of the primary studies. Given the heterogeneity in types of VR intervention and outcomes, a descriptive synthesis was conducted.

**Results:**

A total of 18 randomized controlled trials were included (10 in stroke subcategory, 8 in PD). CASP grading ranged 9–11, suggesting high methodological quality. All studies concluded that overall VR might be as effective as the conventional training, but more motivating. In some studies, VR was found to have a greater effect, taking the high response to treatment and satisfaction into account.

**Conclusions:**

VR training is suggested as an effective intervention to improve the functional ability in stroke and PD patients. Addition of VR into a rehabilitation program might facilitate patient’s motivation, participation and improvement, as this method was generally well accepted, and the results of trials were promising. The consideration of disorder-specific aspects should take place during the decision-making of VR implementation.

## Introduction

The prolonged life expectancy and increase in the aging population worldwide is associated with higher prevalence of chronic, progressive conditions. Among other, neurological conditions are common and strongly affect the life of a person on multiple levels [1, 2]. However, with modern advancement in healthcare these conditions are often optimally maintained by coordinated multidisciplinary efforts [2].

Stroke and Parkinson’s Disease (PD) represent a significant part of the global burden of neurological disorders [1]. Despite the fact that the nature of these two central nervous system disorders is different, with stroke being a cerebrovascular accident while PD is a neurodegenerative disorder, the condition of all patients usually continues to deteriorate over time if not maintained with therapy. Problems with upper and lower extremity functional mobility, balance and coordination, walking and physical capacity are observed in the majority of neurological patients, resulting in decreased functional ability and independence. Cognitive deficits are common among ageing post-stroke and PD patients, affecting social participation and quality of life [1, 3, 4].

The key goal of rehabilitation for the patient groups mentioned above is the maintenance of the functional ability and regain of participation, that is achieved through physical, cognitive and psychosocial improvement. Based on the variety of outcomes contributing to the functional ability, it is believed that the rehabilitation program for a neurological patient should consist of interventions that addresses multiple outcomes [3, 4]. Rehabilitation for persons post-stroke or with PD is often a lifelong process and therefore it is essential that it is patient-centered, accessible, efficient and satisfying [1].

Among other conventional rehabilitative approaches, repetitive task training is important to improve functional independence and locomotion in neurological patients [5, 6]. Continuous task-specific practice is vital for producing and maintaining changes in motor learning, motor function, as well as cognitive state [7]. However, based on observational data, the patients generally perform a limited number of movement repetitions (i.e. low dose) during conventional therapy sessions and spend the time between sessions passively. The lack of motivation for the therapy is often a problem among people with neurological disorders [3, 5]. Possible explanations for this lack of motivation may be these patients’ perception that therapy solely aims to facilitate an adaption to the condition but not full recovery from it, or perhaps lack of encouraging feedback from a therapist. Other logistical, financial, environmental and personal barriers may limit the efficacy of the traditional physical therapy and adherence to long-term rehabilitation plans [5, 6]. As a potential solution for some of the barriers, various digital (*or e-health*) methods are gaining popularity in the neurorehabilitation field, as an adjunct to conventional therapy. Among the numerous advantages and possibilities, it could potentially improve access to therapy, patient engagement in alternative interventions, optimize the efficiency of a therapy session, increase the dosage of task-specific practice, facilitate home-based training and reduce treatment costs [8–10]. One of such proposed methods is the Virtual Reality training.

Virtual reality (VR) is a relatively novell technological concept that is nowadays widely implemented in healthcare [11]. Among other disciplines where it has been adopted, the VR-based rehabilitation is emerging as a distinctive scientific domain in the past 15 years. The clinical implementation is rapidly following discoveries in science and technological advances. According to Keshner [12], the introduction of innovations is so rapid, that the proof on intervention efficacy in different patient populations and possible concerns for research are provided in a more reactive than proactive manner. However, most of the quantitative and qualitative evidence on VR is promising and positive, increasing the interest of clinicians.

The approach of Virtual reality in neurorehabilitation is innovative, as it provides a simulated training of functional tasks at a higher dosage than conventional therapies [4, 13]. It challenges the user for problem-solving and mastering skills in real-life situations in a virtual environment, thus providing the space for harmless failing and learning. In VR, the user receives a visual feedback of the virtual environment and actions through a headmounted device, flat screen or projection system [13]. Feedback can also be provided through other senses, such as hearing, smell, touch and vestibular system. The interaction with a virtual environment is performed by use of a joystick, mouse, sensors, camera, haptic device or other hardware [14]. In the past five years the refinement of the technology resulted in the availability of authoring tools for clinicians to program the virtual environments, as well as the personalization option that adapts the levels of difficulty based on user’s performance. The VR software and hardware has become broadly available due to low-cost and accessibility, introducing the intervention to a variety of clinical and home settings [12].

Previous reviews have stressed the study design and methodological weaknesses of research in the field of VR for neurorehabilitation. The findings and implications were inconclusive, and suggested further research [3, 4, 6, 13]. The majority of the reviews synthesized the articles that were published with up to a ten year difference (eg. compare the findings of 2007 and 2017 trials), included mixed methods studies with lower levels of evidence (eg. compared the findings of observational studies and experimental trials). However the field of telerehabilitation and VR is rapidly developing and innovative solutions have been introduced for previously existing limitations [11]. In such case the earlier conclusions and implications might be irrelevant for nowadays demand and supply.

Providing the evidence for the therapeutic effectiveness of an intervention is important for the validation of VR as an alternative rehabilitation tool for neurological patients. However, it is also important to address the prerequisite skills and factors for benefiting from a technology [15]. These aspects are referred to in this review as “usability”. According to the International Organization for Standardization (ISO), *Usability* refers to “the extent to which a product, a system or a service can be used by specified users to achieve specified goals with effectiveness, efficiency and satisfaction in a specified context of use” (ISO/IEC 9241–11) [16].

Besides the conclusions on effectiveness provided in primary studies, clinically relevant conclusions on implementation in particular patient groups are needed. In other words, it is important for both scientific and clinical communities to understand the aspects of usability, limitations and considerations in the application of the technology.

The aim of this systematic review was to update the existing knowledge with the recent evidence on the effects of VR training on the outcomes contributing to the functional ability of patients with stroke and Parkinson’s Disease. A further aim was to explore whether this intervention is usable for these specific neurological populations.

## Material and methods

This systematic review was conducted in accordance with PRISMA guideline [17] to guarantee high quality reporting. The Cochrane Handbook [18] for systematic reviews was used as a reference for methodology. The protocol of this study was not registered.

### Search strategy and sources

The search strategy was developed and refined after a preliminary search during the project planning. The search took place from September 1, 2019 until February 29, 2020 in PubMed and Cochrane Central Register of Controlled Trials (CENTRAL). The combinations of the following keywords were used: “virtual reality”, “VR”, “stroke”, “Parkinson’s disease”, “PD”, “neurological”, “rehabilitation”. Filters “clinical trial”, “5 years”, “English” were applied. The whole search process consisted of two steps – each diagnosis was searched separately, for example (“virtual reality” OR “VR” AND “stroke”), forming a subcategory of findings as “Stroke”. Each subcategory was searched and analyzed independently. There was no separate search query for the sub-question on VR usability.

To supplement the database search results, the additional records were obtained through manual search of reference lists of the relevant reviews. All results were imported in End Note X9 for screening.

### Eligibility criteria

The studies were included if they met the following eligibility criteria:

#### Type of participants

Studies involved adults (> 18 years), with diagnosis of stroke (both ischemic and hemorrhagic), or PD in chronic or subacute stage. No restrictions were made on gender or disease severity. Participants with other neurological diagnoses or in acute stage were excluded.

#### Type of intervention

Studies with VR as experimental intervention were included, regardless the type of immersion, training location (clinic or home), both exergaming (entertainment purpose, designed for general population) and serious gaming (personalized, task-specific designs for rehabilitation).

#### Type of outcome

There were no strict criteria for the outcomes specified, however they were deemed to be relevant to functional ability or functional independence. Therefore, different outcomes of motor function (upper and lower extremity, gait, balance), cognitive function and quality of life (QoL) were included.

#### Study design

Randomized Controlled Trials (RCT), Randomized Controlled Pilot Trials (RCPT) with minimal sample size of 20. Control group could be any (other intervention, conventional, placebo). Trials with three groups (VR/ other intervention/ control) were also included. Studies were excluded if they applied a “hybrid” approach in experimental group, such as VR with robot-assisted devices or electrical stimulation.

Other study designs, publication language other than English, specific research fields other than physical rehabilitation (occupational therapy, cognitive therapy, etc.) were excluded.

### Study selection

The study selection was performed independently within two subcategories “stroke” and “PD”. Studies were not compared by relevance or methodological quality between the subcategories.

The results of database and reference search were screened by title and abstract relevance to the research question and eligibility criteria. Once a comprehensive list of abstracts was retrieved and reviewed, all studies that met the inclusion criteria were reviewed in full-text. Filtered suitable full-texts were then assessed on the methodological quality of primary studies. In case of similar quality grading, the most recently published studies were included to avoid repetitions of already published systematic review findings in the field.

### Quality assessment and risk of bias

The Critical Appraisal Skills Programme (CASP) checklist for randomized controlled trials was used for the quality assessment of selected studies. The maximum score is 11, including the questions on study design validity (randomization, blinding, group equality at the baseline), reporting of results (sample size/ power calculations, drop-out rates, outcome measures, statistical analysis, treatment effect, potential bias, etc.) and generalization of results. However, the authors of the checklist suggest using it as a tool for subjective analysis of each RCT based on the questions, and not to use it as a strict scoring system [19]. Due to the type of intervention, it was initially assumed that double-blinding was not possible, therefore single-blinding was decided to be sufficient.

### Data extraction and analysis

A data extraction sheet was developed in order to systematically collect data of interest, such as general characteristics (first author, year published, study design), participant information (amount, mean age, type/ stage of disease), intervention description (experimental, control, trial duration and training frequency), investigated outcomes and test measures, treatment effect (within and between group comparison, follow-up) and limitations of primary studies. If no average for some data was presented, the calculations were performed by one of the authors (KS) where possible (eg. mean age, average disease stage, frequency).

### Usability: definitions and concept

The understanding of the usability aspects in this review is based on the concepts and definitions of ISO (ISO/IEC 9241–11). The objective of evaluating the product or interactive system (such as VR) for usability is to enable the potential users to achieve their goals effectively, efficiently (user performance) and with satisfaction (user experience), taking into account the context of use (rehabilitation). Usability is defined as an outcome of interaction, not an attribute of the product, therefore specific attention is paid to personal experiences and circumstances (i.e. particular patient population and their prerequisite skills and limiting factors).

Definitions of the three main components of usability concept:

- *Effectiveness* – “accuracy, completeness and lack of negative consequences with which users achieved specified goals”
- *Efficiency* – “resources (productive time efficiency, human efforts, unnecessary actions, fatigue, accessibility, cost and materials) used in relation to the results achieved”
- *Satisfaction* – “cognitive, psychomotor and affective responses of a user (such as positive attitudes, emotions and/or comfort) resulting from a use of a product or system”.

The concept of usability is proposed as widely applicable in all aspects of product use: accessibility to wide range of users; feasibility for efficient user experience; learnability for skill acquisition; maintainability as adherence and compliance to the product, etc. Therefore the abovementioned aspects, among the other relevant ones extracted from the heterogeneous primary studies with varying terminology are defined by the umbrella-term “usability/ aspects of usability” in this review [16].

The methods for evaluation of usability are not standardized and suggest to include the following content in the analytical inspection of potential usability barriers:

- User observation

- Qualitative: Observation of user behavior and experience
  Quantitative: Measurement of the user performance and responses to obtain data on effectiveness and efficiency

b) Obtainment of user subjective information

- Qualitative: Problems, opinions and impressions of user experience with a product/ system
- Quantitative: Measures of user satisfaction or perception [16].

All content, relevant to the aspects of usability data (whether subjective or only observational) that was possible to extract from the primary studies was analyzed to identify potential problems and barriers associated with the use of such interactive system, as VR, in a context of neurological rehabilitation.

## Results

### General

#### Search results

**Fig. 1:**
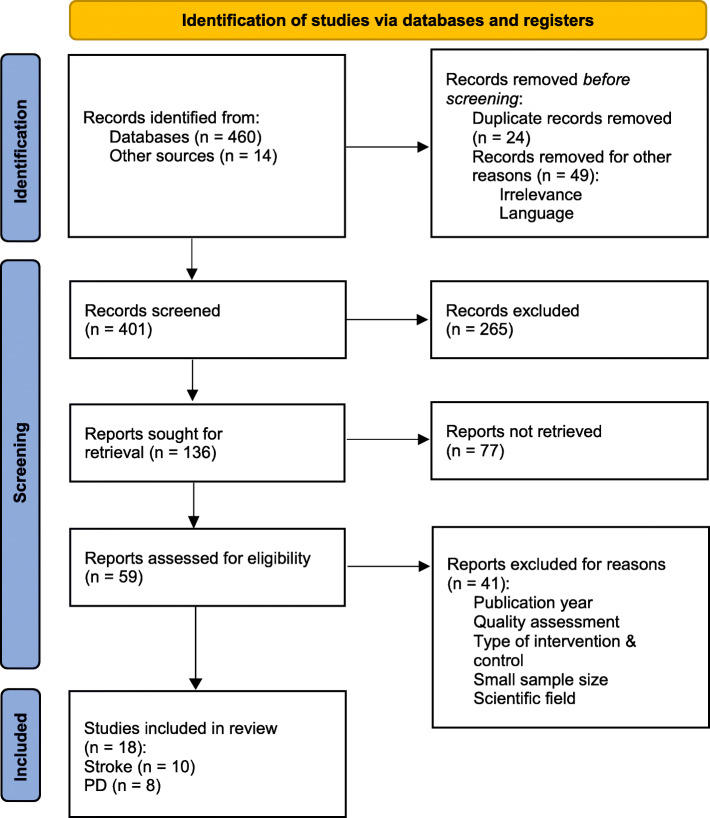
PRISMA flowchart for search results

The initial database search resulted in 460 articles; 14 additional ones were obtained via reference search. Initial selection resulted in the removal of duplicates (*n* = 24) and irrelevant records (*n* = 49) before screening. Following the abstract screening of 401 reports, 136 were sought for retrieval, from which 77 articles were not accessible or available in full-text. Retrieved 59 articles were selected for a full-text reading and assessed by the eligibility criteria. During the full-text reading, some articles were excluded due to the type of intervention (eg. robot-assisted with VR), field of research (eg. occupational therapy, neurocognitive), and small sample size (< 20). Based on the eligibility and quality assessment, 18 articles were finally selected and analyzed in this review. Figure 1 represents the search strategy and results.

#### Quality assessment

Filtered and selected for a full-text reading articles (*n* = 59) were assessed for methodological quality by the CASP tool for randomized controlled trials [19]. Articles included in the final list for the review were graded 9–11 (high quality), assuming that double blinding was not possible in such experimental studies. The grading was not affected if the RCT was at least single-blinded. Five studies [20–24] did not provide a sample size/ power calculations but this limitation was not determinant in the grading. All studies reported on correct randomization procedures, low drop-out rates and few losses to follow-up. Few studies had selective reporting of effects for some secondary outcomes. All studies had limited generalizability of results.

#### Population

In total, 1052 subjects with mild to moderate conditions participated in the reviewed studies, distributed into the two subcategories (stroke, PD). The mean age of all participants of included studies ranged from 55 to 74 years. In all included studies, the experimental and control groups had no significant between group differences at baseline and were treated equally.

Exclusion criteria in most of the trials was severe stage of disease, cardiovascular comorbidities, severe cognitive limitations, visual or hearing deficit, epilepsy, pacemaker device (intervening with VR systems like Wii). In most studies (14 out of 18) the score of a Mini-Mental State Examination (MMSE) was used as an eligibility factor for adequate understanding of procedures and voluntary consent, while two studies on stroke [22, 25] particularly used Montreal Cognitive Assessment (MoCA) as a baseline cognitive assessment tool instead. More detailed information on patient characteristics is described below under subcategories.

#### Interventions

Interventions in experimental groups consisted of certain Virtual Reality modality only (*n* = 12), or combined with conventional training (*n* = 6). The combination of VR and conventional training was applied mostly in stroke trials. Two main types of VR interventions were exergaming and serious games. Among subcategories, exergaming was applied more often than serious games (five out of eight trials) in PD. In the subcategory of stroke, it was even (five trials for each type of VR). Exergaming software and systems were similar across the studies, while serious games were uniquely specific in each study. A summary of interventions among the two subcategories is presented below.

##### Exergaming

Nintendo Wii Fit games with Wii Balance Board [21, 26–28]; Kinect Xbox, Kinect Sports, Adventures, Your Shape games, treadmill [22, 24, 29, 30]; Wii Nintendo Sports – canoe paddling [31], golf, bowling, baseball, tennis [25], jetski, football.

##### Serious games

SeeMe projected video-capture VR system placed in front of treadmill for dual-tasking [20]; VR Rehabilitation System program – Reinforced Feedback Virtual Environment method [32]; Kinect Xbox, KineLabs exergames for stroke (cleaning, cooking) [33]; YouGrabber (Bi-Manu) trainer – sensor gloves [34, 35]; semi-immersive VR system Nirvana – motivating real life environment with multisensory stimulation [36]; scenarios for upper/lower extremity mobility, balance, gait training [23], indoor/outdoor daily activities on balance board and touchscreen VR [37].

##### Control

Conventional physical therapy targeting general functional ability and physical capacity, as well as the particular outcomes, such as balance, gait training, upper and lower extremity mobility, fine movements, cognitive training and neurodevelopmental facilitation. Patient education, stretching and ADL training was also a part of control activities.

In one study [29] the control group was using conventional training combined with robot-assistive device (Armeo Spring) for the upper extremity, for a comparison of two new technologies (VR or robotics as an addition to conventional training).

The duration of the experiment and frequency of the training varied widely between and within the subcategories. The duration of the experiments (in weeks) and the frequency of training (times per week) are described in detail in the subcategories below. None of the trials concluded with the optimal dosage and frequency recommendations. All included studies suggested for further research to determine the duration of a single training session, however this question was not the aim of this review. Therefore, such data was not presented and analyzed here.

#### Results on usability – response to treatment

The patient satisfaction and positive response to Virtual Reality interventions were consistent among studies. It was found to be a fun, motivating, exciting activity compared to usual exercising. Some studies particularly assessed the response to treatment [22, 28, 36] by use of post-intervention ad hoc questionnaires, diaries and feedback surveys, as well as adherence rates while the other studies made conclusions based on verbal patient feedback, involvement and participation during the intervention, drop-out rate or improvement in psycho-emotional state.

### Disorder-specific summary of findings

#### Stroke

In the subcategory of Stroke, ten RCTs were selected and included in the review. In total, 715 stroke (both ischemic and hemorrhagic) patients, mean age range – 55-67 years, participated in trials. Four trials recruited patients in chronic stage [20, 22, 33, 34], four during subacute stage [25, 29, 31, 35] and two trials with both combined [21, 32]. Table 1 presents the characteristics and findings of included studies in stroke subcategory.

**Table 1:** Descriptive characteristics and findings of included studies in stroke subcategory

		Sample	Intervention	Comparison	Outcome	Test	Results	Conclusion	User feedback / follow-up info
[29]	Adomaviciene 2019 RCT	N=42 Subacute Mean age= 64.6	VR Kinect + conventional 2 weeks 5 times/ week	Conventional with robot-assisted trainer “Armeo Spring” 2 weeks 5 times/ week	UE mobility Function* Psycho-emotional	FMA, MAS BBT, HTT ROM, FIM HAD	No between group difference in FIM, but p<0.05 in self-care in VR. UE function significant improvement p<0.05 in both groups VR p<0.05 in HAD	Both groups improved in function, UE mobility and cognitive abilities.	Great user satisfaction, improved psycho-emotional state in VR/ No follow-up
[20]	Fishbein 2019 RCT	N=22 Chronic Mean age= 65.2	VR dual task walking 4weeks 2 times/ week	Conventional treadmill single task walking 4weeks 2 times/ week	Gait Balance Function	10MWT, TUG FRT, BBS ABC	VR p<0.01 in BBS, FRT, 10MWT, ABC	VR is effective in improvement of balance, gait and function. Advised combination with conventional training with multitasking	Follow-up 4 weeks – effect maintained
[32]	Kiper 2018 RCT	N = 136 Chronic, subacute Mean age= 63.9	VR + conventional 4 weeks 5 times/week	Conventional 4 weeks 5 times/week	UE mobility Function	FMA FIM NIHSS ESAS	VR + conventional p<0.05 in all outcomes	VR combined with conventional has greater effect on UE function	No follow-up
[33]	Askin 2018 RCT	N=40 Chronic Mean age= 54.9	VR Kinect + conventional 4 weeks 5 times/week	Conventional 4 weeks 5 times/week	UE mobility Function	FMA, MAS BBT, MI ROM	VR p<0.05 in all outcomes Between group difference VR p<0.05 in FMA, MI, ROM	VR as an effective addition to conventional therapy for UE function and ROM improvement	Good response to VR, great user satisfaction/ No follow-up
[31]	Lee MM 2018 RCT	N= 30 Subacute Mean age= 61.6	VR Wii + conventional 5 weeks 3 times/week	Conventional 5 weeks 3 times/week	UE function Balance	MFT FRT	Both groups p<0.05 in all outcomes. Between group difference p<0.05 in VR in balance, UE function	VR is effective for postural balance and UE function if combined with conventional	No follow-up
[34]	Schuster-Ampf 2018 RCT	N = 54 Chronic Mean age = 61.2	VR 4 weeks 4 times/week	Conventional 4 weeks 4 times/week	UE function Dexterity QoL ADL	BBT CAHAI SIS BI	Both groups p<0.05 in BBT, CAHAI, SIS No between group difference in all outcomes, except for SIS p<0.05 in VR	VR as an effective alternative to conventional therapy in UE function, ADL, QoL. Groups improved more in first 2 weeks.	Greater improvement and response to VR in less impaired / No follow-up
[21]	Utkan-Karasu 2018 RCT	N=23 Chronic, subacute Mean age= 63.2	VR Wii 4 weeks 5 times/week	Conventional 4 weeks 5 times/week	Balance Function	BBS, FRT FIM, TUG	Both groups p<0.05 in all outcomes Between group difference VR p<0.05 in BBS, FRT, FIM	VR is an effective additional intervention for improvement of function, balance, independence	Follow-up 4 weeks – effect maintained
[22]	Lee HC 2017 RCT	N= 47 Chronic Mean age= 57.6	VR Kinect + conventional 6 weeks 2 times/week	Conventional 6 weeks 2 times/week	Balance ADL QoL Satisfaction, feasibility	BBS, FRT, TUG BI, ABC SIS	Both groups p<0.05 in BBS, TUG No between group difference in other outcomes	VR combined with conventional is effective for balance training	Great user satisfaction in VR/ Follow-up 3 months – effect maintained
[35]	Brunner 2017 RCT	N=112 Subacute Mean age= 62	VR 4 weeks4 times/week	Conventional 4 weeks 4 times/week	UE mobility Function ADL	ARAT BBT FIM	Both groups p<0.01 in all outcomes No between group difference	VR as effective as conventional for UE function. Entertaining alternative to standard rehabilitation	Great user satisfaction in VR/ Follow-up 3 months – effect maintained
[25]	Adie 2017 RCT	N=209 Subacute Mean age= 67.3	VR Wii 6 weeks 7 times/week	Conventional 6 weeks 7 times/week	UE mobility Function QoL Cost-effect	ARAT MRS SIS EQ-5D-3L	Both groups p<0.05 in ARAT, EQ 5D 3L No between group difference	VR not superior than conventional, but exciting. Cost-effect - more expensive than home exercise.	Good acceptability of VR/ Follow-up 6 months – no between group difference, but improved health state and arm function

*Function here refers to general functional ability, motor function by functional assessment tools. The terminology varies between the studies

***Abbreviations*:** *10MWT* 10 meter Walk Test, *ABC* Activity-specific BalanceConfidence scale, *ARAT* Action Research Arm Test, *BBS* Berg Balance Scale, *BBT* Box and Block Test, *BI* Barthel Index, *CAHAI* Chedoke McMaster Arm and Hand Activity Inventory, *ESAS* Edmonton Symptom Assessment Scale, *EQ-5D-3L* Quality of Life measure, *FIM* Functional Independence Measure, *FMA* Fugl-Meyer Assessment, *FRT* Functional Reach Test, *HAD* Hospital Anxiety and Depression scale, *HTT* Hand-Tapping Test, *MAS* Modified Ashworth Scale, *MI* Motricity Index, *MFT* Manual Function Test, *MRS* Modified Rankin Scale, *NIHSS* National Institute of Health Stroke Scale, *ROM* Range of Motion, *SIS* Stroke Impact Scale, *TUG* Timed Up and Go test, *UE* upper extremity

#### Outcomes & tests

The most common outcome measures were upper extremity (UE) mobility (*n* = 7), general functional ability (*function* in table) (*n* = 9) and balance (*n* = 4). Other also included activities of daily living (ADL) (*n* = 3), quality of life (*n =* 3), arm dexterity (*n* = 1) and cognitive function (*n* = 2). Test choice for the mentioned outcomes was consistent in the majority of studies. The most common tests used were Box and Block Test (BBT), Fugl-Meyer Assessment (FMA) for upper extremity (UE) mobility and function; Functional Independence Measure (FIM) for ADL and general functional ability; Berg Balance Scale (BBS), Functional Reach Test (FRT) and Timed Up and Go (TUG) for balance. Quality of life was assessed by Stroke Impact Scale (SIS) and EQ. 5D3L, which are stroke-specific tools.

Some of the studies [22, 25, 29, 33, 35] also assessed the response to treatment, patient satisfaction, feasibility and cost-effectiveness by post-intervention/ follow-up surveys.

#### Duration & follow-up

The minimum intervention duration was two weeks [29], maximum six weeks [22, 25], and the most common was four weeks. Training frequency varied from two to seven times per week, the most common was four times per week. Total number of sessions varied from 8 to 42, with most common 16–20 sessions. Based on the study findings, the interventions with longer duration (i.e. 4–6 weeks) generally resulted in stronger, gradually increasing improvements. Five studies had follow-ups, two at four weeks [20, 21], two at three months [22, 35] and one at six months [25]; all with persisting effects.

#### Effectiveness

Half of the trials showed no significant between group differences, but improvements post-test compared to pre-test in UE mobility, functional ability and balance (*p* < 0.05) were demonstrated in both the experimental and control groups [21, 22, 25, 29, 35]. In all trials that assessed balance and functional ability, the experimental group (VR or VR combined with conventional) showed a greater treatment effect (p < 0.05). The same tendency for improvement in the experimental group was observed in five trials for UE function, independence and quality of life [20, 31–34]. No adverse events, associated with the trials were reported.

### Parkinson’s disease

In the PD subcategory, eight RCTs with a total sample of 337 persons were included. Trials recruited patients with all severity stages of PD, on average Hoehn & Yahr (H&Y) 2–3. Mean age ranged from 61 to 74 years. Table 2 presents the characteristics and findings of included studies in Parkinson’s disease subcategory.

**Table 2:** Descriptive characteristics and findings of included studies in Parkinson’s disease subcategory

		Sample	Intervention	Comparison	Outcome	Test	Results	Conclusion	User feedback/ follow-up info
[36]	Pazzaglia 2020 RCT	N=51 Mean age= 71	VR (Nirvana) 6 weeks 3 times/week	Conventional 6 weeks 3 times/week	Balance UE function QoL Satisfaction	BBS, DGI DASH SF36	VR p<0.05 in all outcomes Conventional only p<0.05 in DASH Between group difference in satisfaction, fatigue VR p<0.05	VR more effective than conventional for improvement in function and QoL in safe and stimulating environment	Great user satisfaction in VR/ No follow-up
[26]	Santos 2019 RCT	N=45 Mean age= 64.3	1.VR Wii 2.VR+ conventional 8 weeks 2 times/week	Conventional 8 weeks 2 times/week	Balance Gait Function* QoL	BBS DGI TUG PDQ39	Both groups p<0.05 in BBS, TUG, DGI No between group difference	Combined VR+ conventional has largest effect in all variables. VR as an effective addition to rehabilitation	No follow-up
[23]	Feng 2019 RCT	N= 28 Mean age= 67.2	VR 12 weeks 5 times/week	Conventional 12 weeks 5 times/week	Balance Gait Function	BBS TUG, FGA UPDRS	Both groups BBS,TUG, FGA p<0.05 Between group p<0.05 (VR favor)	VR is promising intervention for balance, gait, mobility. Improved self-care ability	No follow-up
[30]	Ferraz 2018 RCT	N=62 Mean age= 69	VR Kinect 8 weeks 3 times/week	1.Aerobic (bike) 2.Conventional (functional) 8 weeks 3 times/week	Physical capacity Gait Function QoL Depression	6MWT SST 10MWT WHODAS PDQ39 GDS	All groups p<0.05 in 6MWT, SST, WHODAS VR p<0.05 in 10MWT, PDQ39 No between group difference	VR improves walking capacity in PD. All 3 interventions improved gait, functionality	No follow-up
[24]	De Melo 2018 RCT	N=37 Mean age= 62.3	VR Kinect 4 weeks 3 times/week	1.Treadmill 2.Conventional 4 weeks 3 times/week	Gait Function Physical capacity	6MWT Borg scale sp02	VR p<0.05 in 6MWT, Borg No between group difference in VR and treadmill	VR improved walking speed, distance, temporal gait variables, less fatigue. Not proven as effective as treadmill for physical fitness. Combination suggested	VR perceived enjoyable, encouraging/ No follow-up
[27]	Ribas 2017 RCPT	N=20 Mean age= 61	VR Wii 12 weeks 2 times/week	Conventional 12 weeks 2 times/week	Balance Fatigue Function QoL	BBS FSS 6MWT PDQ39	VR p<0.05 in BBS, FSS No between group difference in functional capacity	VR is effective in enhancing balance, reducing fatigue after 12 weeks	Follow-up 4 weeks with doing only ADL exercises - no long-term effect
[28]	Gandolfi 2017 RCT	N=71 Mean age= 68.7	VR Wii (at home) 7 weeks 3 times/week	Conventional (at clinic) 7 weeks 3 times/week	Balance ADL Gait Cost-effect Satisfaction	BBS, DGI ABC 10MWT	Both groups p<0.05 in DGI,ABC,10MWT Between group difference VR p<0.05 for BBS, conventional p<0.05 for DGI.	VR (with carer) is feasible alternative to in-clinic VR has lower treatment and equipment cost	Same level of satisfaction in both groups/ Follow-up 4 weeks – effective.
[37]	Yang 2016 RCT	N= 23 Mean age= 74	VR 6 weeks 2 times/week	Conventional 6 weeks 2 times/week	Balance Gait Function QoL	BBS, DGI TUG UPDRS PDQ39	Both groups p<0.05 in all outcomes No between group difference in any outcome	VR as effective as conventional in balance, motor, gait, QoL. Interesting addition to home program.	Follow-up 2 weeks - no between group difference.

*Function here refers to general functional ability, motor function by functional assessment tools. The terminology varies between the studies

***Abbreviations*:** *6 MWT* 6 minute Walk Test, *10MWT* 10 meter Walk Test, *ABC* Activity-specific Balance Confidence scale, *BBS* Berg Balance Scale, *DASH* Disability of Arm, Shoulder and Hand Q, *DGI* Dynamic Gait Index, *GDS* 15-item Geriatric Depression Scale, *FGA* Functional Gait Assessment, *FSS* Fatigue Severity Scale, *PDQ39* 39-item Parkinson Disease Questionnaire, *SF-36* The Short Form Health Survey, *SST* Sit-to-Stand Test, *TUG* Timed Up and Go test, *UPDRS* Unified Parkinson Disease Rating Scale, *WHODAS* WHO Disability Assessment Sscale

#### Outcomes & tests

The most common outcome tested was balance (*n* = 6), gait (*n =* 6), general functional ability *(function* in table*)* (*n =* 6) and quality of life (*n* = 5). Other outcomes included physical capacity (*n* = 2), ADL (*n* = 1), UE function (*n =* 1), depression (*n =* 1) and fatigue (*n =* 1). Test choice for the mentioned outcomes was consistent in a majority of studies. The most common tests used were BBS, Dynamic Gait Index (DGI) for balance; TUG, 6-min Walk Test (6MWT), 10-m Walk Test (10MWT) for gait and functional ability. Quality of life was mostly assessed by PDQ39, a Parkinson’s-specific tool.

Some of the studies [28, 30, 36, 37] also assessed the response to intervention, patient satisfaction, feasibility and cost-effectiveness by post-intervention/ follow-up surveys.

#### Duration & follow-up

Minimum intervention duration was four weeks [24], maximum twelve weeks [23, 27], most common six or eight weeks. Training frequency varied from two to five times per week, most common was three times per week. Total number of sessions ranged from 12 to 60, most common 12 or 24 sessions. Interventions with longer duration (i.e. 8–12 weeks) generally resulted in gradually increasing improvements that persisted over time if the therapy was maintained. One study had a follow-up at two weeks [37] and two studies at one month [27, 28]. In one study [27] no long-term effect was observed or reported.

#### Effectiveness

One study [26] found a greater effectiveness of combined VR and conventional training than VR alone on balance, gait and function. No significant between group differences in functional outcomes was shown in five of eight studies [24, 26, 30, 36, 37]. In five studies [23, 26, 28, 30, 37] both experimental and control group pre-post test improvement was found in functional outcomes and balance (*p* < 0.05). Some studies showed significant improvement of VR group in gait, balance, quality of life and fatigue [24, 27, 30, 36], while no effect was seen in the conventional training group. A significant between group difference was observed in satisfaction, fatigue [36] and balance [28] in favor of VR. No adverse events, associated with the trials were observed.

## Discussion

### Findings

This review analyzed the effects of Virtual Reality training as a rehabilitation intervention for stroke and Parkinson’s Disease patients. These two common neurological disorders require continuous management in order to maintain functional ability and to improve quality of life through the interventions, targeting multiple outcomes [38, 39]. All studies in this review proposed the VR training to be as effective compared to conventional training. It was found to be advantageous for improvement of the functional abilities in neurological patients, namely upper extremity functional mobility, balance, gait, as well as cognitive, psychoemotional aspects and quality of life. Based on these findings, VR might be suggested for an inclusion in a neurorehabilitation program as a beneficial addition to conventional therapy in patients with mild to moderate conditions. However the treatment effect in most of the trials was only short-term. The findings of trials that included a follow-up assessment [20–22, 25, 28, 35, 37] suggest that effect could be maintained if patients continued training for a longer time (i.e. longer than four to eight weeks, which was the most common duration), taking into account the adaptation to new intervention, technology acceptance and motor learning. Based on the characterstics of included trials, no conclusions could be drawn on the optimal frequency of the training or the session duration, suggesting that the dosage and frequency should be tailored to a specific patient’s condition and capacity, with a potential progression. Since VR is emerging within the telerehabilitation field as an activity performed at home, gradual implementation of it to the patient’s home environment may result in continuous adherence of training and potential long-term effects on functional outcomes [28].

The decision to include a broader scope of study population was reasoned by the aim to explore the consistency of findings (and as a result have better generalizability) across different types of participants facing similar functional deterioration over time [18]. In some way it may counterbalance the fact that nearly one third of included RCTs had small sample sizes and lacked generalizability in their conclusions. Despite the rapid growth of telerehabilitation and Virtual Reality research fields, there is still a relatively small amount of quality evidence for PD population. Previously published VR trials and reviews in PD compare the findings to other diagnoses, due to insufficient data within the same population. This review found some consistency in outcomes and test choice among trials in both populations (eg. BBS, TUG were the most common tests among both subgroups) and therefore it is believed that common conclusions could be drawn.

### Types of virtual reality

Another important distinction to be discussed, is the Virtual Reality approach – exergames and serious games. Exergames are the commercially available games designed primarily for general healthy population and accessible for anyone having the equipment at home or in clinic. Due to a wide variety of scenarios and intensity levels it can be successfully applied in the home-based telerehabilitation of neurological patients [40]. Serious games however, are specifically designed for rehabilitative purposes for a particular limitation, are less entertaining and more task-oriented. The potential limitation of exergames is that their complex interfaces may not be compatible with postural or mobility constraints, or can be cognitively challenging for a person with a disability [41]. Confusion and disappointment of being unable to use the system can result in non-compliancy. Flexibility and task-orientation of the virtual reality games is therefore imperative, based on a qualitative patient experience review of Lewis and Rosie [42]. It was found that adult neurological patients are seeking more rehabilitative benefits of therapeutically principled design, rather than just playing games [43, 44]. On the other hand, exergames can be perceived as a more enjoyable, fun activity that is distracting from daily problems, since it does not distinguish the patient with special needs from otherwise healthy users. It is also easier to obtain cost- and availability-wise, to vary games and to use it together with caregiver or family members for some social interaction [28, 44].

The type of VR was not a determinant factor for the study inclusion, however interesting tendencies were observed between the subcategories. Coincidentally, the distribution of both approaches among the stroke subcategory trials was even. The VR research in stroke is the largest among other neurological populations, and it might explain the wide variety of approaches investigated. In the PD subcategory, the exergaming as an experimental intervention was slightly more common than serious games (five vs three trials).

It might be assumed that serious games are more valuable for a targeted rehabilitation due to the specifically designed task-oriented scenarios for the patients with limited capacity. On the other hand, the preferrence for serious games might be disadvantageous due to higher costs associated with the design and implementation, as well as the limited availability.

### Usability

In order to decide whether the intervention is usable for the particular population, three points of interest need to be analyzed – its effectiveness, efficiency and satisfaction, according to the ISO definition of usability of the product or interactive system [16]. An important point to mention, is that the terminology (many synonyms used) and definitions (wide use in relevant aspects/ context) within this complex concept are all interrelated and interdependent and should be analyzed together as a whole. Some aspects that are more commonly addressed or can be quantitatively assessed and evaluated such as response to treatment, satisfaction, adherence and cost-effectiveness are emphasized more in studies. It can be used as a basis for deeper analysis of other aspects that are less commonly addressed or could not be quantitatively evaluated.

Based on the results of the systematically reviewed trials, some conclusions could be drawn on the effectiveness of the intervention (due to the connection between the two aims of this review) and patient satisfaction (possible to evaluate). However the topic of efficiency is left open, which was thought to be particularly important to discuss from a clinical point of view.

The definition of “*Efficiency” (see Methods section)* includes the factor of human efforts for productivity. Some accompanying deficits and practical barriers resulting in increased human effort and subsequent decreased productivity might affect the successful introduction of this potentially beneficial rehabilitation tool into a neurological patient’s daily routine. Therefore the aim of this review was not only to analyze the effectiveness of VR on functional outcomes, but also to explore whether this innovative intervention is usable for the mentioned neurological populations. There was no separate search query on the aspects of usability due to a limited amount of relevant evidence, therefore the discussion is based on the characteristics of the reviewed trials with a support of the literature.

#### Patient satisfaction

The positive response to treatment and perceived enjoyment of the VR therapy, leading to the improved quality of life was mentioned in a majority (10 out of 18) of included studies. Several trials that particularly assessed user satisfaction, concluded the significant improvement in mood, motivation and psychoemotional state in favor of VR [22, 28, 36]. Other authors based their conclusion on the maintainability (adherence and low drop-out rates, positive verbal patients’experiences and interest in continuation with VR) after the experiment [24, 25, 29, 30, 33–35]. These conclusions are in line with a number of previous reviews covering the topic of VR user satisfaction [3, 4, 40].

A common problem in conventional physical training is the lack of motivation [3, 5]. However it is vital to keep a patient motivated during the long-term rehabilitation to optimize the training outcome, prevent frustration and habituation [45]. Interesting suggestions were made by Perez-Marcos et al. [45], that neurorehabilitation programs should be inspired by Seligman’s „PERMA “theory (i.e. positive emotions, engagement, relationships, meaning, achievement) [46] and create an experience of the patient being comfortabilly challenged and engaged by the task which renders high levels of enjoyment.

According to Lewis and Rosie [42], the key components that make VR enjoyable for patients are the challenging environment, replication of real-life situations, sense of control and success. Engaging, entertaining activities should be incorporated in neurorehabilitation program for a patient to be willingly participating in their own recovery and be responsible for independent training at home, especially in case of life-long rehabilitation [45]. Worth to mention is that a majority of the included trials (15 out of 18) reported low or zero drop-out rates and great adherence, suggesting the positive maintainability and engagement in the intervention. Due to the fact that the effectiveness of both conventional training and VR was similar in a majority of trials, combining these two interventions might result in a better adherence to long-term plans of care [22, 24, 26].

#### Sensory deficit and VR

Visual and hearing deficits are very common among the general aging population and can be influenced by a comorbid condition. During the review analysis, it was noted that in a majority of included trials (12 out of 18) the visual or hearing deficits were an exclusion criteria for study participants [21–24, 26–28, 30, 33–36]. While it might be apparent to not include patients with sensory deficits since it affects the ability to adequately follow instructions and overall performance during the VR training, it raises a concern for the selective accessibility and usability of such intervention. All mentioned studies pointed out this fact as a study limitation affecting the generalizability of the results. Congruent multisensory environments stimulating somatosensory system (eg. via proprioception) besides visuomotor coordination need to be proposed [47]. However, there is an on-going innovative development of VR specifically for visually-impaired persons, with enhanced auditory stimulation, or with haptic feedback through cane controller [48].

#### Cognitive deficit and VR

Another exclusion criteria in all 18 trials was a severe cognitive deficit that was either tested during the screening process using the assessment tools (MMSE, MoCA) or just mentioned as an eligibility criteria without a test in two studies [21, 24]. Unfortunately, cognitive decline is common among the studied population too, due to general aging and deteriorative changes in the brain due to a disorder [41]. In contrast with the visual deficit that cannot be altered with the use of Virtual Reality, the cognitive training however became a separate branch within the VR field [49, 50]. Traditional cognitive practices are often directed towards the isolated cognitive domains, such as executive functions, attention, visuospatial ability, memory and language. However cognition can be trained multidimensionally through the real-life situation scenarios simulated via Virtual Reality, potentially resulting in a sustained improvement of functional independence in activities of daily living. Thus it may be assumed that VR settings are more ecologically valid while there are no real consequences of failure [50].

The cognitive deficit can negatively impact the recovery of functional ability and quality of life. Therefore, to increase the therapy efficiency, the cognitive training should be incorporated into the motor VR training [45]. The functional training in virtual environment would have an influence on cognition through multitasking, dynamic feedback processing and progressive learning. In this review, some included trials introduced a dual-task activity in the experimental group [20, 22, 28, 30] or assessed the indirect effect of VR on cognition [29, 34]. Dual-task paradigms are a powerful way to evaluate the capacity of divided attention on individual task (cognitive task while performing motor activity) [51]. For example, dual-tasking is associated with gait impairments and results in freezing of gait, decreased stride length and symmetry loss among the general PD population. It does not improve with the dopamine replacement therapy [51], therefore should be addressed by combined physical and cognitive interventions. Serious games using principles of motor learning and neuroplasticity can optimize recovery after brain damage, such as stroke [41].

#### Aging and technology

Technology acceptance has been a topic of attention since the emergence of telemedicine systems and the introduction of the telerehabilitation [8, 52, 53]. Regardless of the benefits and effectiveness of a certain telerehabilitation tool (eg. VR), its feasibility depends on whether the potential users have sufficient access and skills to use the digital technology [10, 54]. It is assumed that acceptance and understanding of VR software and hardware is greater among younger patients, as they might be more familiar with the use of digital technologies [52, 55]. Persons that acquire the disability such as stroke or PD in a relatively older age, might face some challenges with the implementation of digital tehcnologies in their daily life [56, 57]. Decreased learning capacity is augmented by the resistance to change, low level of confidence, lack of skills for using the technologies, and different expectations based on traditional rehabilitation approaches [42, 54, 56]. Sometimes an unsuccessful implementation of VR can be explained by the negative preconceptions of the older person. According to Laver [58], some patients preferred the conventional therapy due to a social interaction with staff, or biased beliefs that VR is too childish and provides only entertainment. However, some publications had positive conclusions on the coping strategies for adoption of digital technologies among older patients regardless their prior level of technological familiarity [53, 54].

The concerns for a technological acceptance described in reviewed research are mostly relevant for the current decade when it was published (i.e.2010–2020), the period of rapid growth of the telerehabilitation field and the introduction of innovative solutions. However it is expected to change dramatically over the next 10–20 years. The percentages of active users are likely to increase together with further development of digital services. The implementation of e-health technologies and its awareness is increasing, subsequently resulting in more technologically literate people among the retired [59]. Current 40–60 years old active users will eventually become the potential users of telemedicine tools, during some transition stage of their life [60].

#### Adverse effects

No adverse effects were reported in included trials. The participants in all trials had an opportunity to register their adverse effects associated with the experiment in order to drop-out, while diaries and questionnaires were used to monitor it in four studies [22, 25, 28, 36]. However, according to some previous research [61, 62], some adverse effects might be observed in such populations and therefore should be enlightened.

VR provides the patient with the real-life situations in virtual environment, increasing the sense of own abilities. Immersive VR gives the “sense of presence”. This however might provoke hallucinations in neurological patients. Hallucinations can occur in different neurological conditions due to affected sensory processing but is particularly frequent among Parkinson’s disease patients [63]. In such neurodegenerative disease like PD, the hallucinations might be explained by a combination of factors – sensory loss and dysfunction in modulatory mechanisms, dream intrusion phenomena and pharmacological side effect of the dopaminergic treatment [62, 63]. The hallucinations and impaired adaptation to visuomotor perturbations typically occur in an off-medication state during or after the VR session [4, 62]. It might lead to a suggestion to provide VR training only in an on-medication state and add the topic of hallucinations to the patient anamnesis. In this review, all included studies in PD subcategory had the trainings in “on” state and had short follow-up period, what might be a reason why no potential adverse events were reported or discussed. Other negative impact such as “cybersickness” (nausea, dizziness, headache, cold sweat), eyestrain and associated fall-related injuries were reported for both neurological patients and general population interacting with VR technology [61, 64]. The patient’s feedback and well-being should be monitored during and after the session for safety reasons. In case of home-based independent use of VR, the safety precautions must be taken [4, 65].

### Strengths and limitations

The strength of this review is that it provides the analysis of the recently published high-quality evidence on effectiveness of VR on functional abilities of patients with stroke and Parkinson’s disease. It also discusses the usability of such intervention in the mentioned neurological populations. The review overviews the possible barriers for successful introduction of VR intervention, such as disorder-related deficits and technology acceptance of aging population, in order to help the decision-making of a health professional whether this tool is suitable for a specific patient’s case.

Some limitations in this review could affect the overall quality of conclusions. Main limitation is the heterogeneity of included primary studies with regards to the type of virtual reality (immersion levels, software, serious vs exergames), as well as the treatment duration and frequency. The review also does not provide the in-depth analyses of particular VR interventions, its’ technological structure and content. It is, however, worth to mention that the review did not aim to investigate a specific type of VR for the most effective type, or finding an optimal duration of the training program. The focus was rather the general effects of VR therapy on functional abilities of a patient and the usability aspects of the implementation of VR into rehabilitation programs of selected aging populations. The heterogeneity is explained by inclusion of several outcomes, as well as by continuous innovation in VR market. Another limitation is a small sample size in a number of included primary studies that influence the generalization of findings. However, the majority of studies in this relatively new research field possessed a small sample, so only high methodological quality articles were selected for inclusion. Unfortunately, due to the nature of intervention it was not possible to blind the patients during the trials in primary studies. Therefore, despite the high methodological quality of included articles, none of them followed the double-blinding procedure.

### Recommendations for further research

According to the limitations that were faced while conducting this review, several recommendations for future primary and secondary research are suggested. There is a demand for high methodological quality RCTs with larger samples (especially in the PD field), as well as standardized outcome measures for the most common functional outcomes in order to increase the generalizability of the results.

As this review emphasizes the need of overviewing the aspects of usability of the new technological intervention, it is strongly recommended to include the assessment (in terms of standard questionnaires, diaries, etc.) and analysis on usability/ feasibility/ applicability/ patient satisfaction. It would then result in a higher quality systematic reviews able to quantitatively analyze the actual usability aspects and draw more valid conclusions.

There is also a number of additional questions of interest for future studies to focus on, for example: the optimal duration and frequency of training; correlation of training duration and new skill acquisition; comparison of VR exergames and serious games content and usability; efficiency of VR as an online tool for home-based training compared to an in-clinic supervision and other potential aspects that could not be covered in this review.

## Conclusion

Virtual reality training is suggested to be as effective intervention to improve the functional ability in stroke and Parkinson’s disease patients as the conventional training. Adding the Virtual Reality training to a rehabilitation program might facilitate the improvement of upper extremity functional mobility, balance, gait, activities of daily living, quality of life, psycho-emotional state and cognition. Motivational and exciting VR training results in high patient satisfaction and engagement. This intervention is usable for stroke and Parkinson’s disease patients, if disorder-specific deficits and technological concerns are taken into account before participation. The Virtual Reality training is suitable in-clinic and as a telerehabilitation tool at home, if safety precautions are followed.

## Data Availability

Available from authors.
